# Viral Involvement in Alzheimer’s Disease

**DOI:** 10.1021/acschemneuro.0c00719

**Published:** 2021-03-09

**Authors:** Ahmad Sait, Cristian Angeli, Andrew J. Doig, Philip J. R. Day

**Affiliations:** † Division of Evolution and Genomic Sciences, Faculty of Biology, Medicine and Health, The University of Manchester, Manchester M13 9PL, United Kingdom; ‡ Manchester Institute of Biotechnology, The University of Manchester, Manchester M1 7DN, United Kingdom; § Faculty of Applied Medical Science, Medical Laboratory Science, King Abdulaziz University, Jeddah 21589, Saudi Arabia; ∥ Division of Neuroscience and Experimental Psychology, School of Biological Sciences, Faculty of Biology, Medicine and Health, The University of Manchester, Manchester M13 9PT, United Kingdom; ⊥ Department of Medicine, University of Cape Town, Cape Town 7925, South Africa

**Keywords:** Alzheimer’s disease, herpes simplex virus, β-amyloid, valacyclovir, blood−brain barrier, apolipoprotein E

## Abstract

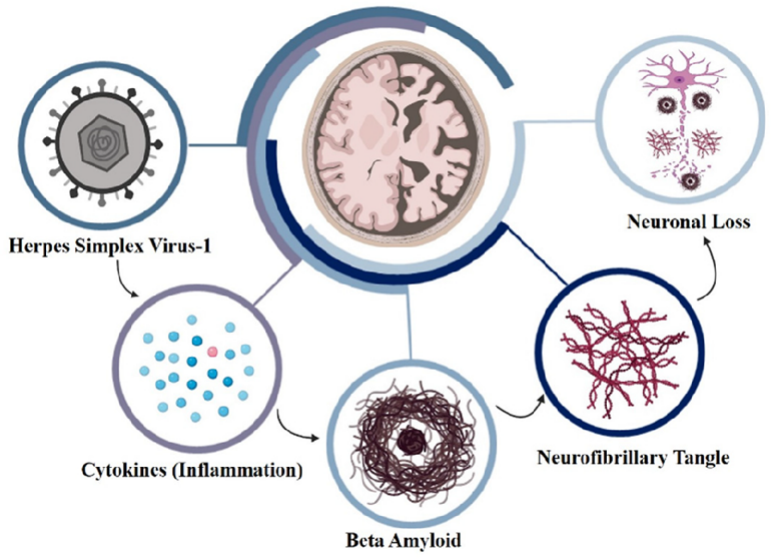

Alzheimer’s disease (AD) is characterized by the presence of β-amyloid plaques (Aβ) and neurofibrillary tangles (NFTs) in the brain. The prevalence of the disease is increasing and is expected to reach 141 million cases by 2050. Despite the risk factors associated with the disease, there is no known causative agent for AD. Clinical trials with many drugs have failed over the years, and no therapeutic has been approved for AD. There is increasing evidence that pathogens are found in the brains of AD patients and controls, such as human herpes simplex virus-1 (HSV-1). Given the lack of a human model, the route for pathogen entry into the brain remains open for scrutiny and may include entry via a disturbed blood–brain barrier or the olfactory nasal route. Many factors can contribute to the pathogenicity of HSV-1, such as the ability of HSV-1 to remain latent, tau protein phosphorylation, increased accumulation of Aβ *invivo* and *in vitro*, and repeated cycle of reactivation if immunocompromised. Intriguingly, valacyclovir, a widely used drug for the treatment of HSV-1 and HSV-2 infection, has shown patient improvement in cognition compared to controls in AD clinical studies. We discuss the potential role of HSV-1 in AD pathogenesis and argue for further studies to investigate this relationship.

## 1 Introduction

Dementia is the decline of brain function, including memory impairment, causing loss of ability to perform daily activities. It is usually linked to aging. In 2015, around 47 million people worldwide were affected, with more than half of the cases of dementia being Alzheimer’s disease (AD).^1,2^ Symptoms of AD include progressive memory loss, then loss of ability to speak and eat.^3^ The typical duration of this disease is 8–10 years.^4^

AD was described by the German psychiatrist Alois Alzheimer in 1906. It is a neurodegenerative disease, with associated loss of neurons in the brain that produce deterioration of memory and cognition.^5^ AD has two main subtypes: first, a sporadic type or late onset Alzheimer’s disease (LOAD) and, second, early onset Alzheimer’s disease (EOAD) which starts before the age of 65. The most important pathological markers for all classes of AD are the accumulation in the brain of β-amyloid plaques (Aβ) and the formation of neurofibrillary tangles (NFTs).^6^

AD is a multistage progressive neurological condition affecting cognition, behavior, and mood, with stages that are complex to characterize. The majority of AD research focuses on pathogenic processes most often related to accumulation of Aβ and tau proteins. A thorough detailed mapping of AD pathogenesis will better establish disease mechanisms including those that may associate AD with pathogen infections.

To date, there is no treatment for AD.^7^ Many clinical trials failed to remove Aβ in AD patients, and none of them have reduced the pathogenesis of AD.^8^ Currently approved drugs can only alleviate symptoms, such as cholinesterase inhibitors (donepezil, rivastigmine, and galantamine) that act by slowing the breakdown of acetylcholine in the synaptic cleft and memantine, an antagonist of glutamatergic NMDA receptors.^9,10^ Despite enormous academic and commercial research investments, an effective treatment for AD remains elusive; ergo a different research approach is compelling. Over the past 30 years, research investigations have steadily revealed a potential role for viruses (Table 1) in AD pathogenesis.^11−13^

**Table 1:** Herpesviridae Virus Family Found in the Brains of Alzheimer’s Disease Patients^a^

virus	*P*-value	number of AD patients	number of non-AD control patients	biological sample	method of detection	percentage positive for AD patients and controls	ref
HSV1	<0.05	21	191	brain tissue	*insitu* hybridization	81% of AD patients	(19)
						47% of controls	
	NA	8	6	brain tissue	PCR	All patients and controls were positive for HSV1.	(32)
	NA	21	15	brain tissue	PCR	67% of AD patients	(26)
						60% of controls	
	<0.0001	39	36	brain tissue	PCR	78% of AD patients	(20)
						64% of controls	
	NA	17	12	brain tissue	PCR	75% of AD patients	(27)
						60% of control	
	0.89	73	33	brain tissue	PCR	74% of AD patients	(31)
						73% of controls	
	0.447	27	13	CSF, serum	ELISA	52% of AD patients	(23)
						69% of control	
HSV-2	NA	53	39	frontal and temporal cortex brain tissue	PCR	13% of AD patients	(16)
						23% of controls	
HHV-3	NA	17	12	brain tissue	PCR	None in AD patients or controls.	(27)
HHV6	<0.003	50	35	frontal and temporal cortex brain tissue	PCR	72% of AD patients	(16)
						40% of controls	
HHV6	NA	27	13	CSF, serum	ELISA	22% of AD patients	(23)
						none of controls	
CMV	NA	45	29	frontal and temporal cortex brain tissue	PCR	36% of AD patients	(16)
						35% of controls	

a NA = not available from the publication.

Here we explore the possibility of HSV-1 involvement in AD pathogenesis. HSV-1 infections are commonplace and the virus has the ability to persist in a latent form within neurons of their human host. While many HSV-1 infected individuals remain asymptomatic and some of these may not progress to develop AD, this review discusses those other cases where HSV-1 could be a causative agent for AD.

## 2 Herpes Simplex Virus-1

HSV-1 is a neurotropic virus that infects 90% of the world’s population and causes oral ulcers. Infection with HSV-1 usually occurs during the first years of life and can then become latent for long periods of time.^14,15^ The Herpesviridae family has many species. Those found in humans include (A) herpes simplex virus 1-2 (HSV-1-2), (B) human alphaherpesvirus 3 (HHV-3), known also as varicella-zoster virus (VZV), (C) human herpesvirus 6 (HHV-6), and (D) cytomegalovirus (CMV) (Table 1).^16^ The most common viral strain which is found in the brain of AD patients and linked to AD pathogenesis is HSV-1.^17^

## 3 HSV-1 in the Brains of AD Patients

Growing evidence links HSV-1 to AD pathogenesis: First, HSV-1 DNA has been found in AD patients, localized within amyloid plaques in temporal and frontal cortices.^13^ Numerous studies were performed on AD patients to investigate the presence of HSV-1 (Table 1). HSV-1 was found to be present at significantly different levels (Table 1) between the brains of AD patient and control groups.^19,20^ Underlying pathogenesis mechanisms appear to make only a subpopulation of AD patients positive for HSV-1. Therefore, HSV-1 may not be a causative agent by itself but may play a role in the pathology of AD through interactions with the host. Microbial pathogens, like HSV-1, can not only cause an acute infection but can also remain in a latent phase^21,22^ HSV-1 is more likely to be active in elderly people,^23^ suggesting that HSV-1 may reactivate after latency. In addition, the immune response of the host to a microbial infection will vary from person to person.^22^

The first Koch postulate is that microbes should be found in abundance in all organisms suffering from the disease and not in healthy individuals. However, Koch later recognized that some microbes could be present in patients without any symptoms.^24,25^ Infection therefore does not mean that the patient will always be (phenotypically) affected^22^ and therefore does not preclude HSV-1 from being associated with the development process of AD.

In a study conducted by Dealty et al., 17 of 21 patients with AD were positive for latent HSV-1 RNA in the trigeminal nerve (where HSV-1 can establish latency), while 59 of 191 controls were positive for latent HSV-1 RNA in the trigeminal nerve using in situ hybridization (*P* < 0.05).^19^ Jamieson et al. found that HSV-1 might be localized in many parts of the brain including temporal lobes, frontal lobes, and the hippocampus.^26^ HSV-1 DNA was found in 14 of 21 AD patients and 9 of 15 controls, using PCR to detect the thymidine kinase gene of HSV-1. In the same study, temporal and frontal tissues from 10 controls (newborn and middle-aged) were tested for HSV-1 and all samples were negative. This finding suggests that the elderly are be more likely to carry HSV-1.^26^

Itzhaki et al. found that AD patients with the HSV-1 DNA have a higher presence of the APOE4 allele than in a HSV-1 negative AD group, HSV-1 negative non-AD group, and HSV-1 positive non-AD group (52.8%, 10%, 6.3%, and 3.6%, respectively) with respect to allelic frequency of APOE4 in each group.^20^

Lin et al. conducted a study on the presence of *Varicella zoster* virus (VZV) and HSV-1 in AD patients. Twenty-four samples were taken from 17 AD patients and 20 samples from 12 controls.^27^ VZV DNA was negative in all samples, but 17 of the AD patient samples and 12 of the control samples were positive for HSV-1.^27^ It appears that latent HSV-1 in the CNS is found in both AD patients and normal controls, with VZV absent in the CNS. There are two possible explanations for this variance. First, the source of the CNS infection could be from exogenous or endogenous virus. In HSV encephalitis, infection might be exogenous with HSV-2 reinfection or endogenous by reactivating from latency in the peripheral nervous system (PNS).^28,29^ In VZV, the reactivation occurs just once and only several months after immunosuppression, where HSV-1 can reactivate from 1 to 4 weeks of immunosuppression. Second, if the source of the CNS infection is exogenous, a possible route is via the olfactory bulb.^27^

Liedtke et al. tested for the presence of HSV-1 and VZV in the olfactory bulb in post-mortem samples from humans, finding that HSV-1 is present in 15 out of 97 samples, whereas VZV was present in only 1 out of 97 samples.^30^ HSV-1 is therefore more likely to enter the brain via the olfactory bulb than VZV, increasing the chances of a recurrent infection for HSV-1.^27^

Beffert et al. investigated the presence of HSV-1 and APOE4 allele in 73 AD individuals and 33 controls. 74% of AD patients and 73% of controls were positive for HSV-1. There was no significant difference in APOE4 presence between patients with and without HSV-1 (*P* < 0.19)^31^ which contrasts with other findings.^20^ Other research carried out by Wozniak et al. on the presence of intrathecal antibodies against HSV-1 and HHV6 in 27 AD patients and 13 control subjects showed that 52% of AD patients and 69% of controls were positive for HSV-1,^23^ and there was no significant relationship between IgG levels of HSV-1 in patients with AD and controls (*P* = 0.447).^23^ The presence of IgG indicates that HSV-1 can cause acute infection in both control and AD patients other than in the case of herpes simplex encephalitis. Nevertheless, it is unknown whether the presence of IgG reflects the initial arrival of the virus in the brain or whether it is more likely that the virus is subsequently reactivated unnoticed.^23^ For HHV6, belonging to the same family as HSV-1, 22% of patients with AD were positive and none of the controls had antibodies against HHV6, which may be due to the lack of infection in CNS or a limited immune response.^23^

Lin et al. conducted a study of 148 AD patients and 103 controls to detect the presence of DNA from HHV6, HHV2, and CMV. Their results showed that HHV6 was present in 72% of AD patients and 40% of controls with a significant difference (*P* < 0.003), while for HHV2 and CMV there was no significant difference between AD patients and the control group (Table 1).^16^

## 4 Importance of the Blood–Brain Barrier

The blood–brain barrier (BBB) is an extremely selective semipermeable membrane that separates the peripheral bloodstream and other cellular fluids from the brain. It consists of endothelial cells, pericytes, astrocytes, and tight junctions.^33^ The primary function of the BBB is to provide a stable environment for the central nervous system (CNS) to function by allowing glucose and amino acids to cross while preventing microbial pathogens from passing through the BBB.^34^ A dysfunctional BBB can be seen in the tissues of many diseases, including brain tumors, multiple sclerosis, Parkinson’s disease, and AD. An increased BBB permeability may allow bacteria and viruses to enter the brain.^35^ Sometimes the increased permeability can be exploited by permitting drugs into the brain, such as penicillin in the case of meningitis.

The receptor for advanced glycation products (RAGE) accumulates in aging cells and is found in even higher amounts in AD.^36^ Low density lipoprotein receptor-related protein 1 (LRP-1) is accountable for brain homeostasis.^37^ RAGE and LRP-1 are responsible for the clearance of Aβ from the brain. In AD, the expression levels of these molecules change leading to the transit of leukocytes and amyloid proteins across the BBB (the gastric abdominal and BBB become more penetrable with aging).^38^ Dysfunction of the BBB may impact AD, as the clearance rate of Aβ can be lowered, causing accumulation of Aβ in the brain and triggering the amyloid pathway.^39^

## 5 Possible Routes for HSV-1 To Enter the Brain

**Figure 1:**
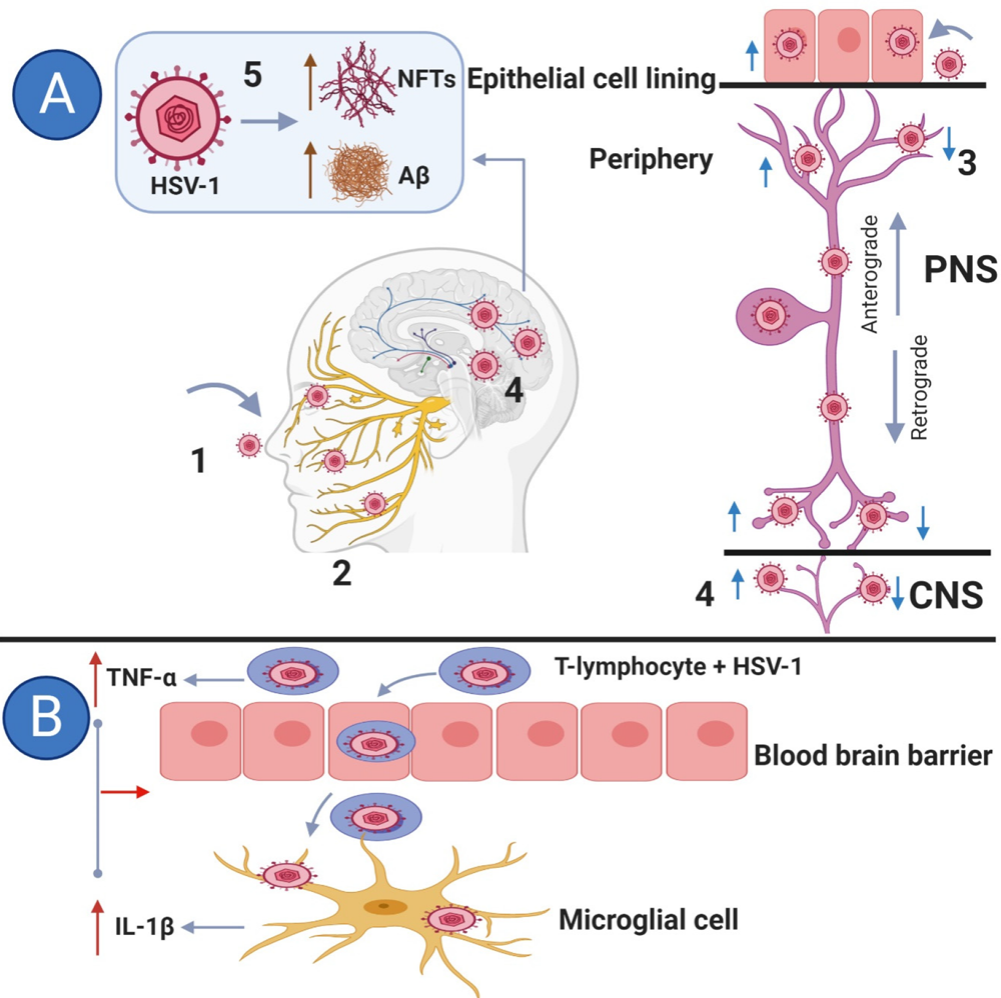
Possible routes for HSV-1 to enter the brain. Blue arrows indicate the virus movement. (A) (1) A suggested main route for HSV-1 to enter the brain is by infecting the epithelial cell line in the nasal cavity, causing a cold sore. (2) The virus can remain latent in the trigeminal nerve for an extended period of time. (3) Reactivation of the virus if immunocompromised and during chemotherapy. (4) The virus travels back to the primary site of infection and causes further cold sores and can reach the central nervous system (CNS) via sensory neurons in the peripheral nervous system (PNS). (5) HSV-1 induces the accumulation of β-amyloid plaques (Aβ) and neurofibrillary tangles (NFTs) inside the brain. (B) The virus can use T lymphocytes to cross the blood–brain barrier (BBB). The infection of the T lymphocyte with the virus stimulates the production of TNF-α and increases the production of interleukin-1β (IL-1β) in microglial cells, resulting in the breakdown of the BBB. Created with BioRender.com.

There are two main routes suggested for HSV-1 to enter the brain (see Figure 1). First (Figure 1A), the virus infects the epithelial cells in the oral or nasal mucosa, followed by olfactory bulb conduction to reach autonomic ganglia, at which point the HSV-1 becomes latent and thus evades the immune system. Under the conditions of stress, immunodeficiency, or chemotherapy, the virus reactivates and infects (PNS) neurons. At this stage, the capsid is transported to reach the primary site of infection and to initiate replication.^24,40,41^ The virus can reach the CNS through sensory neurons which have pseudo-unipolar axons, traversing epithelial cells and synapses linking into CNS neurons.^41,42^

Second, HSV-1 might enter the CNS directly through blood circulation (Figure 1B). A study performed on mice injected with HSV-1 intraperitoneally revealed that the virus was detected in blood, then subsequently in various brain regions, including midbrain, brain ventricles, cortex, and cerebellum.^42,43^ The midbrain area is an important route for HSV-1 to enter the brain through the blood circulation because of the functional and anatomical relation between adrenal glands and hypothalamic suprachiasmatic nucleus.^43^

The exact mechanisms through which HSV-1 could enter the brain in humans is still highly controversial. Herpes simplex encephalitis (HSE) is a condition in which HSV-1 causes brain inflammation and creates a possible route for HSV-1 to access the brain, which can be via the BBB.^44^ The BBB disruption can result through many mechanisms including aging, hypercholesterolemia, hypertension, smoking, and the action of viruses.^45,46^ Once the BBB is disrupted, which is often the case in AD patients, then HSV-1 might enter the brain by penetrating immune cells, such as T-lymphocytes or macrophages to cross the BBB.^17,46^ As shown in Figure 1B, HSV-1 infected macrophages can infiltrate the BBB through release of tumor necrosis factor-α (TNF-α) and stimulating microglial cells to produce interleukin-1β (IL-1β). These cytokines are essential for the adhesion of endothelial cells and can therefore influence the permeability of the BBB.^17,42^ Marques et al. found that cytotoxic T-lymphocytes may be present in the brains of mice after 14 days of HSV-1 infection.^47^

The route of entry for the HSV-1 to the brain via the BBB might not be possible in humans, as virus migration to the brain by the BBB has only been tested in mice. Also, if the virus was present in the blood, that should cause viremia and complications for the patient, and this is rarely the case in AD.^48^

## 6 Evidence for HSV-1 Reactivation

One of the most important pathological features of HSV-1 is its ability to reactivate under many conditions, including immunosuppression, neurosurgery, and radiotherapy in humans. In addition, the reactivation of HSV-1 was tested in mice by thermal stress.^24,40^ If HSV-1 is activated in the brain, it results in an acute inflammation of the brain known as encephalitis^49^ which can cause severe symptoms such as fever, seizures, abnormal behavior, and loss of consciousness.^45^ Although there is no direct test to measure the reactivation of HSV-1 in the brain, there is evidence that HSV-1 can be reactivated. Lövheim et al. found that IgM against HSV-1 was found in AD patients which indicated a recent reactivation of HSV-1.^50^

HSV-1 was introduced to nine mice by intranasal inoculation, and after 60 days the viral ICP4 protein (which activates transcription during infection) of HSV-1 was found in trigeminal ganglia and the cerebral cortex, with many inflammatory biomarkers present, including interferons (IFN) α/β and Toll-like receptor 4 (TLR4) in trigeminal ganglia and the cerebral cortex.^51,52^ Yao et al. showed that the viral load of HSV-1 in the brain was higher than in trigeminal ganglia in mice infected with HSV-1, suggesting that HSV-1 could reactivate in the brain.^53^

De Chiara et al. reactivated HSV-1 in mice using thermal stress and found an accumulation of Aβ and hyperphosphorylated tau in the neocortex and hippocampus. Interleukin-1β (IL-1β) and interleukin-6 (IL-6) are important mediators of the inflammatory response produced by immune cells and can reactivate HSV-1.^54−56^ These two cytokines were also elevated in the neocortex region in mice infected with HSV-1.^40^

## 7 HSV-1 Contribution to β-Amyloid Plaques in Alzheimer’s Disease

**Figure 2:**
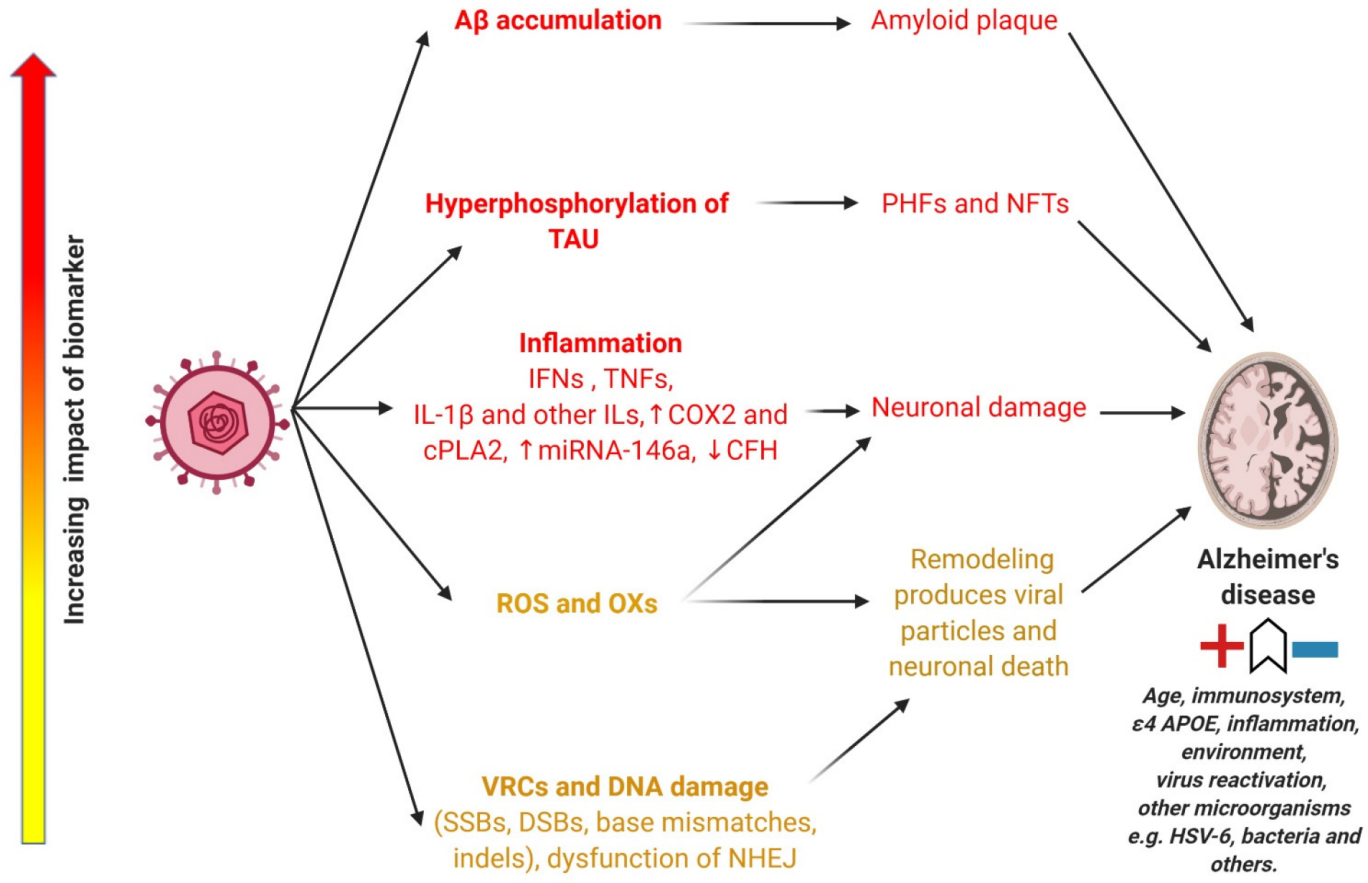
How HSV-1 might cause Alzheimer’s disease (AD). The main suggested routes to pathogenesis have been listed according to their ascending impact (from yellow, amber to red). The red sector shows biomarkers most prevalently associated with AD including Aβ accumulation, hyperphosphorylation of tau, and inflammation. These are related to HSV-1 studies where Aβ accumulation leads to AD through amyloid plaque formation. Hyperphosphorylation of TAU contributes to AD through paired helical filaments (PHFs) and neurofibrillary tangles (NFTs).

There is increasing evidence suggesting that HSV-1 can be linked to the pathogenesis of AD (Figure 2). The accumulation of Aβ in the brain is a well-known characteristic of AD. Aβ may also act as an antimicrobial peptide against bacterial and viral infections.^57,58^ Bourgade et al. examined many cell lines, including fibroblast, neurons, and epithelia, in the presence of Aβ-40 and Aβ-42 in combination with HSV-1, and their results suggest that Aβ-40 and Aβ-42 prevent the replication of HSV-1.^59^ Aβ-42 antiviral activity was measured by infecting human monocytes and neutrophils with influenza A virus (IAV). Aβ-42 reduced viral protein synthesis in monocytes, and no H_2_O_2_ (nothing recognized as a foreign body) was detected in neutrophils.^58^

The glycoprotein B (gB) in HSV-1 has sequence homology to the carboxyl-terminal region of Aβ which interferes with HSV-1 replication.^59,60^ In addition, gB produces β-pleated sheets resembling Aβ fibrils which might accelerate the rate of Aβ deposition into a form that is toxic to primary cortical neurons.^59,60^ A study on transgenic 5XFAD mice infected with *Salmonella typhimurium*, HSV-1, and HHV-6 showed that Aβ peptides could protect the mice against brain infections.^61,62^ Taken together, this evidence suggests that Aβ can be used as an antimicrobial peptide, but the overproduction of Aβ due to the repeated cycle of reactivation for HSV-1 could lead to the accumulation of Aβ in the brain and activate the pathogenic amyloid pathway.^17,49,61^

Inflammation causes neuronal damage through the production of proinflammatory cytokines such as interferons (IFNs), TNFs, interleukin-1β (IL-1β), and other ILs and regulation of inflammation associated molecules such as cyclooxygenase-2 (COX2), cytosolic phospholipase A2 (cPLA2), miRNA146a, and complement factor H (CFH).

In the amber and yellow AD biomarker risk categories, the production of ROS and OXs contributes to AD neuronal damage. HSV-1 replicates by the formation of viral replication compartments (VRCs) and contributes to DNA damage such as single-strand breaks (SSBs), double-strand breaks (DSBs), base mismatches, indels, and dysfunction of DNA repair mechanisms mainly at nonhomologous end joining (NHEJ). These dysfunctions lead to AD by remodeling of the host cell DNA for the production of new viral particles and subsequent neuronal death. The most important risk factors that modulate AD progression include age, immune system function, ε4 APOE, inflammation, environment, and perhaps virus reactivation and presence of other microorganisms.

## 8 Hyperphosphorylation of Tau Protein by HSV-1

**Figure 3:**
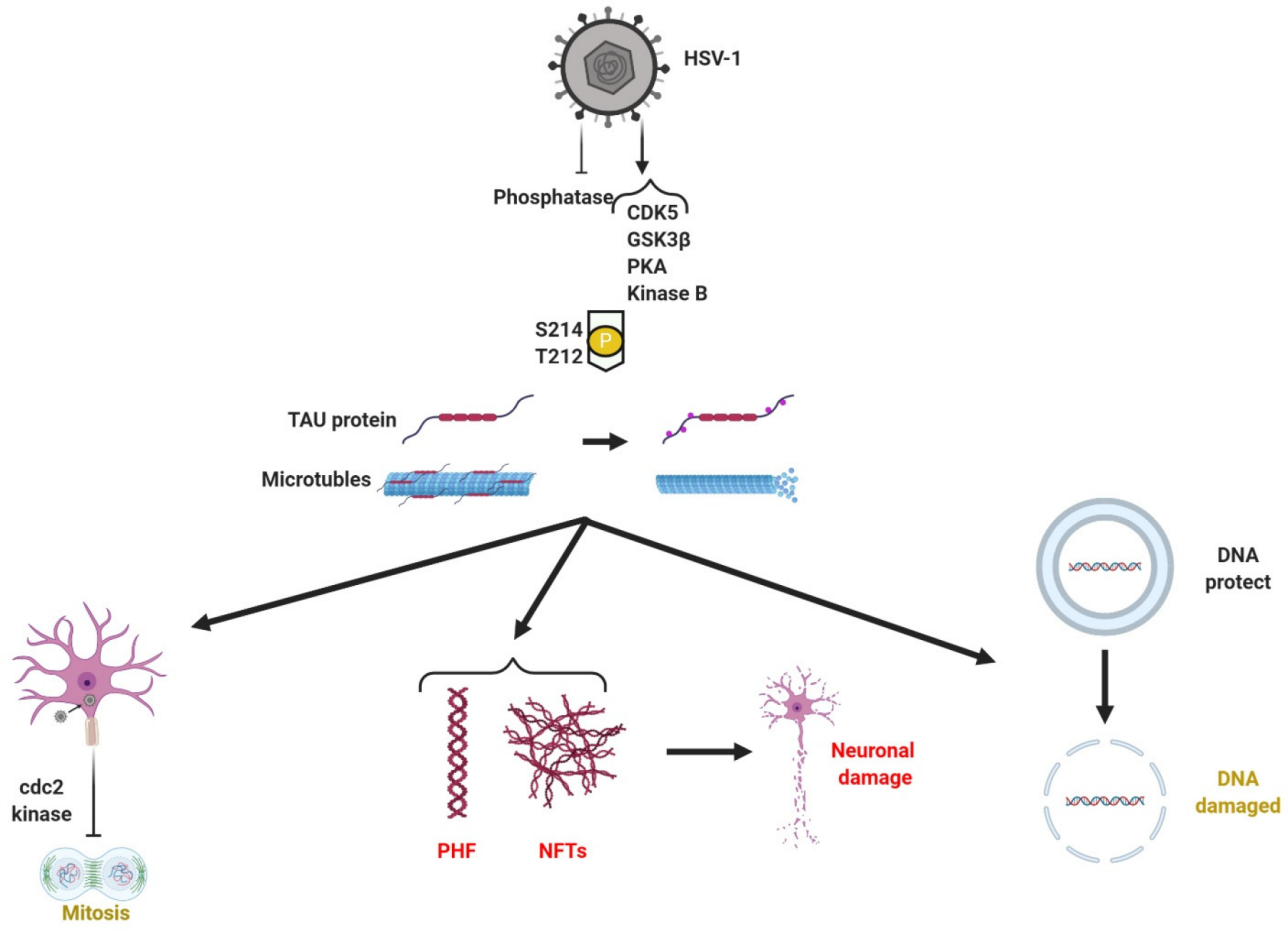
HSV 1 brain infection causes hyperphosphorylation of tau protein leading to Alzheimer’s disease. HSV-1 is able to increase CDK5, GSK3β, PKA, and kinase B activities while decreasing the activities of several phosphatases. These enzymes phosphorylate tau proteins (that normally stabilizes microtubules) at positions S214 and T212, among others. Microtubule structure is lost with phosphorylated tau creating cellular disorder. HSV-1 activates cdc2 kinase blocking cellular mitosis. Then, the formation of PHFs and NFTs leads to neuronal damage and the DNA becomes susceptible to damage. Created with BioRender.com.

Tau protein is a microtubular-associated protein encoded by the MAPT gene on human chromosome 17.^63^ It is mainly expressed in neuronal axons and causes tubulin polymerization for assembly of microtubules used in axonal transport.^40^ When phosphorylated, tau loses solubility and forms filaments associated with neuronal death and neurodegenerative disease such as AD^64^ and tauopathies^63^ (Figure 2). HSV-1 increases the hyperphosphorylation of tau protein, already present in AD patients, mainly at serine–proline (serine 214 or S214 specific for AD) and threonine–proline (threonine 212 or T212 specific for AD) motifs (Figure 3) as shown from the immunohistochemistry work performed on SHSY-5Y cells by Wozniak et al. S214 and T212 are sites where hyperphosphorylated tau is common in AD.^65^

Hyperphosphorylation of tau allows HSV-1 to enter neurons^65^ and permits modulation of host cell cycle machinery (activating cell division cycle protein 2 kinase normally not expressed in resting neurons) and blocks mitosis that otherwise interferes with the viral cycle (Figure 3). HSV-1 can phosphorylate tau in several ways. First, HSV-1 increases the activity of cyclin-dependent kinase 5 (CDK5) in neuroblastoma SK-N-MC cells.^66^ Second, HSV-1 increases the activity of glycogen synthase kinase 3β and protein kinase A (PKA). GSK3β is a multifunctional serine/threonine kinase with the ability to phosphorylate tau and regulate several cell cycle signaling pathways. PKA is able to deliver cellular signals by adding phosphates to several proteins.^67,65^ Third, HSV-1 increases the activity of kinase B (Figure 3) that cleaves TauC3.^68^ HSV-1 is able to decrease the activity of many phosphatases (Figure 3) as shown from immunohistochemistry and immunofluorescence studies performed on SHSY-5Y cells.^65^

Hyperphosphorylation of tau, caused by HSV-1 (Figure 2), leads to the formation of paired helical filaments (PHFs) and NFTs (Figure 3) whose density correlates with disease severity and dementia.^63^ Alvarez et al. used immunostaining to show hyperphosphorylated tau inside the nucleus of SK-N-MC cells to be caused by HSV-1 VRCs. VRCs are the machinery of HSV-1, where viral proteins are produced to assemble new virions.^66^ By increasing tau phosphorylation, HSV-1 is able to spread its virions through the microtubules. Zambrano et al. demonstrated that HSV-1 induced microtubule rearrangement in mice primary neuron cultures which is necessary for viral dissemination to the neuronal nucleus. The increased cytoskeleton stability subsequently facilitated viral exit and with it reduced neuron viability.^64^

Tau protein protects host cell DNA from modification and damage because microtubules (to which tau is bound) are linked to the LINC (linker of nucleoskeleton and cytoskeleton) complex which keeps stable the nuclear envelope^69^ and because tau protein can directly bind AT rich DNA which protects host cell DNA from modification and damage.^70^ When HSV-1 hyperphosphorylates tau, the DNA protection is weakened and the host cell accumulates DNA damage that can lead to cell death (Figure 3).^49,65^

## 9 APOE 4 Allele and HSV-1 in AD

Apolipoproteins (APOEs) are responsible for regulating and carrying lipids in the bloodstream and can be expressed in astrocytes.^41^ APOE3 is the most common allele and seems to play a role in the fibrillogenesis, oligomerization, and clearance of Aβ. APOE4 is involved in lipid transport and is considered an important risk factor for AD (Figure 2) as it has a scarce ability to bind Aβ, so its expression contributes to Aβ accumulation and aggregation within neurons.^41^ A mouse model humanized for the APOE3 or the APOE4 alleles infected with HSV-1 showed that mice with APOE4 have a higher virus load in the brain compared to those with APOE3.^71^ Another study using transgenic mice with APOE knockouts for APOE3, or APOE4 alleles infected with HSV-1, found that in the CNS, APOE knockout mice had a lower HSV-1 level then APOE3 and APOE4 mice. Mice with APOE4 had a higher amount of virus compared to APOE3 mice, suggesting that APOE4 plays an essential role in HSV-1 and AD.^72^

## 10 HSV-1 and Enhanced Inflammation in AD

Brain inflammation is one of most important hallmarks of AD (Figure 2). HSV-1 load is believed to increase inflammation in the AD brain, mainly after reactivation of HSV-1 from the trigeminal ganglion, and in older people with a weakened immune system^52^ and higher permeability of BBB.^73^ The condition is determined by the detection of high levels of cytokines, IgG, and IgM (mainly after HSV-1 reactivation) in the blood of AD patients.^74,75^ The main type of immunity that is activated is the innate immune response (the most rapid defense system for the host cell) to combat HSV-1 infection,^76^ as demonstrated in hispid cotton rat (*Sigmodon hispidus)* displaying brain inflammation and multifocal demyelination caused by HSV-1.^77^

## 11 How Does HSV-1 Activate the Brain Immune System?

**Figure 4:**
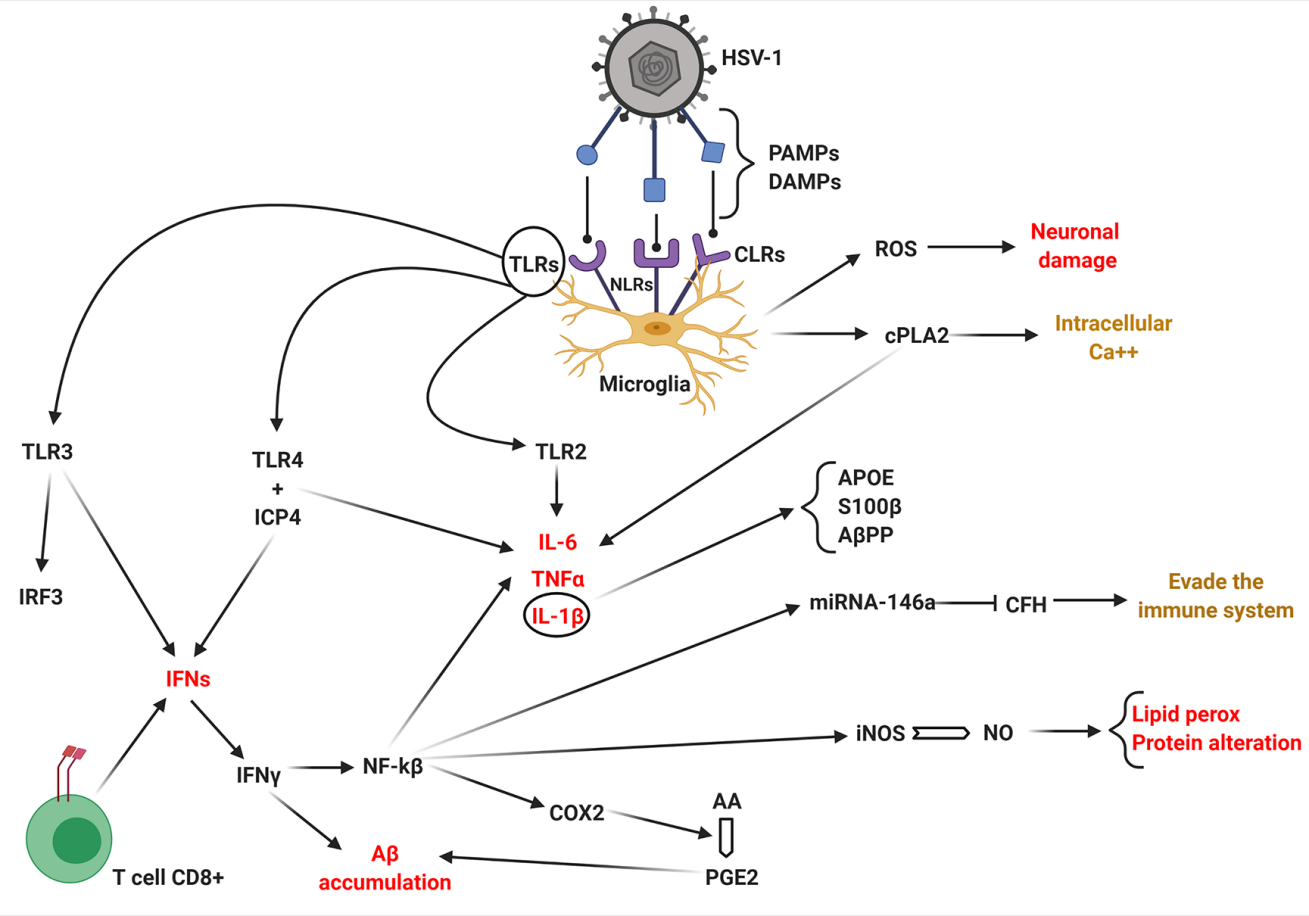
How HSV-1 infection leads to brain inflammation that contributes to Alzheimer’s disease. Microglia are activated by host TLRs, NLRs, CLRs and by virus PAMPs or DAMPs. TLR3 stimulates the production of IRF3 and IFNs (together with TLR4 (via viral ICP4) and T cells CD8+). IFNγ mainly leads to Aβ accumulation through the expression of NF-kβ that upregulates COX2 and AA transformation in PGE2. NF-kβ increases the enzymatic activity of iNOS. Increased production of NO causes lipid peroxidation and protein alteration and expression of miRNA-146a which inhibits CHF and viral escape from the immune system. TLR2 increases the production of cytokines (IL-6, TNFα, IL-1β that stimulate APOE, S100β, and AβPP). Activated microglia increase the production of ROS that cause neuronal damage and the expression of cPLA2 that further stimulates cytokine production and intracellular calcium. Created with BioRender.com.

The neural tissue infection by HSV-1 provokes an innate and adaptive immune response, mainly within microglial cells that are the center of the innate immune response of the brain (Figure 4).^78^ The microglia-mediated immune response largely depends on the pattern recognition receptors (PRRs) that detect pathogen-associated molecular patterns (PAMPs) or damage-associated molecular patterns (DAMPs), which allow microglia to recognize the presence of the virus.^76^ The major microglial PRRs include Toll-like receptors (TLRs). Others are nucleotide oligomerization domain (NOD)-like receptors (NLRs) and C-type lectin receptors (CLRs) (Figure 4).^76^ The work of Martin et al. showed that when HSV-1 infects brain cells (mainly microglia), the TLRs play a major role in CNS inflammation.^52^ An *in vitro* study found increased expression for TLR2, TLR3, TLR4, and interferon regulatory factors 3 (IRF3) and 7 (IRF7). TLR2 is important for the production of IL-1β, IL-6 and TNF-α. TLR3, located in intracellular compartments, has the capacity to sense double-stranded viral RNA (ds RNA), which activates type-1 IFN signaling pathways and the production of cytokines (Figures 2–4). TLR4 is expressed only with the early viral protein infected cell protein 4 (ICP4) that can increase IRF3 and IFNs; for this reason, the authors suggested that the activation of TLR4 in microglia increases the amyloid peptide-induced microglial neurotoxicity (Figure 4) (association with amyloid plaque deposition) and eventual neuronal death.^17,52^

## 12 Which Molecules Are Present during HSV-1 Infection?

The brains of Alzheimer’s disease patients infected with HSV-1 following reactivation show high levels of cytokines and proinflammatory molecules including IL-1β, IL-6, TNF-α, and interferon-γ (IFN-γ) (Figure 2).^73,79^ Proinflammatory molecules upregulated are cyclooxygenase-2 (COX2, a key enzyme in prostaglandin biosynthesis converting arachidonic acid to prostaglandin E2,^80^ that together with cPLA2 is pivotal within the arachidonic acid cascade),^81^ and cytosolic phospholipase A2 (cPLA2), along with miRNA-146a, while complement factor H is downregulated (Figure 2).^81,82^ IL-1β has been show to increase APOE levels and raise astrocyte-mediated S100β activity (a member of the family of S100 calcium-modulated proteins, that acts as a proinflammatory cytokine and as a DAMP molecule; it is neurotropic at low concentration while neurotoxic at high concentration).^83,84^ Amyloid-β protein precursor (AβPP) production and β-site AβPP-cleaving enzyme 1 (BACE-1) (Figure 4) that catalyzes the conversion from AβPP to Aβ^79^ are also increased. HSV-1 is also able to upregulate the expression of miRNA-146a by activating NF-kB^85^ (Figure 4) after which NF-kB stimulates the production of ROS, IL-1β, TNF-α, and Aβ peptide.^86^ The main purpose of miRNA-146a is downregulation of complement factor H (CFH) gene expression^81^ (targeting of the CFH mRNA 3′-UTR by miRNA-146a) (Figure 4). In this way, HSV-1 is free to replicate and the innate immune system is dysregulated.^86,87^ miRNA-146a was found to be upregulated by NF-kB and is abundant in AD brains.^88,89^

cPLA2 is upregulated by HSV-1. It utilizes arachidonic acid-containing phospholipids as the preferred substrate and plays a key role in the initiation of the inflammatory lipid-mediator cascade.^90^ cPLA2 upregulates IL-1β, TNF-α, IFN-γ, and Aβ,^91^ and it stimulates an increase in intracellular calcium (Figure 4).^92^ Several *in vitro* studies have shown that increased intracellular calcium levels can trigger Aβ formation and *vice versa*.^93^

## 13 The Role for IFNs with HSV-1

The production of IFNs through activation of microglial cells and CD8+ T cells (during chronic inflammation) (Figure 4) inhibits viral infections while stimulating the immune system. But when the production is excessive (such as during chronic inflammation), the effects of IFNs on the brain are detrimental.^49,78^ For example, the level of IFN-λ is increased in an AD patient’s brain.^94,95^ The overproduction of IFN-γ increases Aβ accumulation,^96^ and the increase of expression and activity of nuclear factor κB (NF-kβ) (Figure 4) facilitates inducible nitric oxide synthase expression (iNOS, an enzyme capable of generating NO from the amino acid l-arginine).^97^ iNOS increases inflammation within AD brains because it leads to both lipid peroxidation and functional alteration of proteins (these modifications are molecular markers of AD).^98^ NF-kβ activity upregulates cytokine and proinflammatory molecules, such as IL-1β, TNF-α, ROS, and COX2.^92^ The production of PGE2 has been shown to stimulate the production of Aβ (Figure 4).^99^

## 14 HSV-1 Might Cause NHEJ Dysfunction

**Figure 5:**
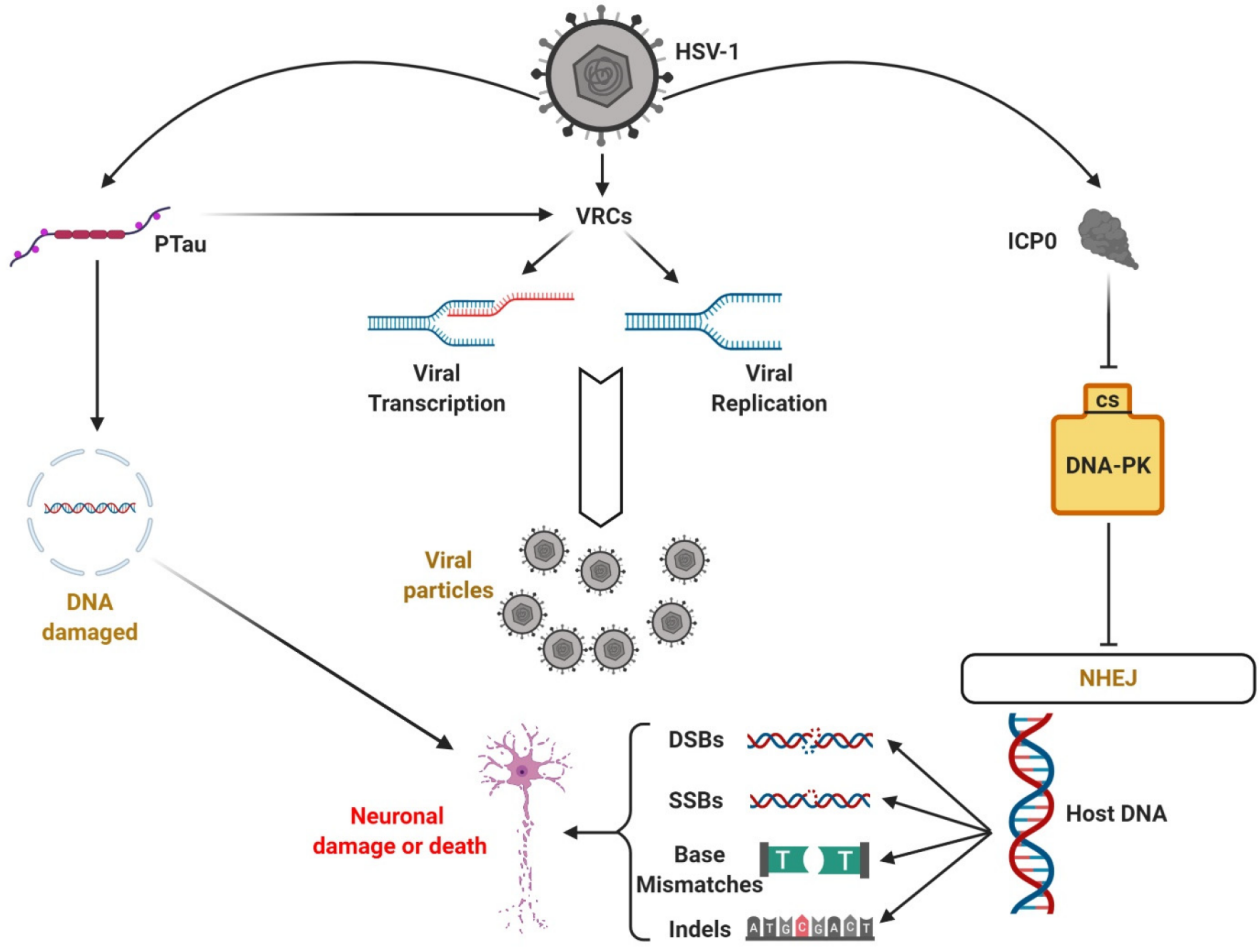
HSV 1 viral replication compartments (VRCs) and DNA damage contribute to Alzheimer’s disease. HSV-1 cellular infection creates VRCs that lead to viral replication and transcription and is further increased following tau phosphorylation (PTau). HSV-1 is then able to inhibit DNA-PK through ICP0 that binds DNA-PKcs. In this way, NHEJ loses function and DNA is susceptible to damage, such as double/single strand breaks, base mismatches and indels, leading to neuronal cell damage and death, which is potentiated through PTau-associated reduced protection of DNA damage. Created with BioRender.com.

HSV-1 contributes to AD through the dysfunction of many DNA repair systems.^100^ One of the most important is nonhomologous end joining (NHEJ) (Figure 2).^49^ NHEJ is a versatile enzyme pathway that mainly repairs double-strand breaks (DSBs) (the most dangerous damage in DNA) by recognition, cutting, and reconstruction of the correct sequence (Figure 2). NHEJ also acts against other damage such as single strand breaks (SSBs), base mismatches, and indels. For DSB recognition, NHEJ use a Ku70/80 heterodimer that is part of another important enzyme involved and recruited by the NHEJ process, a DNA-dependent protein kinase (DNA-PK). DNA-PK is involved in DSB repair (DSBRs) and possesses DNA-PKcs kinase activity (Figure 5).^101,102^

During infection, HSV-1 expresses the gene infected cell polypeptide 0 (ICP0) that is part of the Immediate-Early (IE) genes family, controlling the configuration of the viral genome inside the host cell. ICP0 is also an E3 ubiquitin ligase that induces degradation of the DNA-PKcs (Figure 5).^100,103,104^ In this way, HSV-1 leads to NHEJ dysfunction and apoptosis.^49,105^

## 15 Antiviral Studies of HSV-1 in the Elderly

Valacyclovir is the most widely used antiviral agent for the treatment of HSV-1, HSV-2, herpes zoster, and chickenpox. Valacyclovir works through the conversion of viral thymidine kinase to monophosphate (acyclo-GMP) and triphosphate (acyclo-GTP) forms. The acyclo-GTP acts as an inhibitor of the viral DNA polymerase and selectively kills infected cells without affecting healthy cells.^74^ The levels of tau protein, Aβ, and HSV-1 were reduced in a Vero cell model when using acyclovir, penciclovir, and foscarnet.^106^

A recent cohort study investigated the association between HSV infection and dementia. The results showed that patients with HSV are 2.56 times more likely to develop dementia compared to the control group. The risk was reduced in patients treated with antiherpetic medications, including acyclovir, famciclovir, ganciclovir, idoxuridine, penciclovir, tromantadine, valaciclovir, and valganciclovir.^107^ The use of valaciclovir in people at high risk for AD could reduce the risk of developing AD by preventing HSV-1 from being reactivated.^107^ The main argument here is that the drug only works on viruses that actively replicate and not against latent HSV-1. However, preemptive treatment to avoid HSV-1 activation is unproven and requires clinical approval. There are presently two major clinical studies on AD patients to measure the effect of the valacyclovir drug (NCT02997982 (April 3, 2020) and NCT03282916 (August 2022)).^108^

## 16 Conclusion

Although there is substantial evidence indicating that HSV-1 can be linked to the pathogenesis of AD, the issue is still controversial on whether the association is causative or reactive. For example, if HSV-1 is involved in the pathogenesis of Alzheimer’s disease, then the reactivation of HSV-1 in the brain should be noticed, as the patients ought to suffer from encephalitis. In addition, antivirals are used to treat acute infections (cold sores and encephalitis) and will not be active to remove latent HSV-1. In a recent cohort study,^107^ in which they found that antivirals reduce dementia, viral load was not studied, so there may be some other confounding variables affecting the final results. In addition, antibody studies against HSV-1 could be misleading since the presence of IgG indicates a previous infection rather than a recent one and it could be due to a viral protein rather than an infectious virus.

Indeed, the use of near limit of detection sensitive measurements creates a potential for false lab results which is a real-world concern. It is apparent that not everyone with HSV-1 develops AD, which may be in part due to environment, genetics, and comorbidity. Previous studies found that HSV-1 is present in both AD patients and controls (Table 1), and despite the statistically significant presence of the virus between the brains of AD patients and control (because the brain is typically believed to be sterile), it is compelling to know if there is any variation in viral load between the two cohorts to understand if there is a threshold for the virus to trigger an amyloid biosynthesis pathway in AD patients. Moreover, the viral strain is not assessed in these studies (Table 1), which could give an indication of which strain of HSV-1 is most common in AD patients. The ability of HSV-1 to cause neurodegenerative disease can be seen by (1) inducing accumulation of Aβ and NFTs *invivo* and *in vitro*, (2) phosphorylation of tau protein, (3) causing inflammation, (4) damage to the neuronal DNA, (5) reactivation from latent state. In summary, the evidence suggests that HSV-1 could well play a crucial role in the pathogenesis of AD. More clinical studies and over a sustained period of time are needed for people at high risk of AD to better understand whether the treatment against HSV-1 can prevent or delay neurodegenerative disease.
